# Crystal structure of semi-synthetic obelin-*v* after calcium induced bioluminescence implies coelenteramine as the main reaction product

**DOI:** 10.1038/s41598-022-24117-5

**Published:** 2022-11-15

**Authors:** Pavel V. Natashin, Elena V. Eremeeva, Mikhail B. Shevtsov, Margarita I. Kovaleva, Sergey S. Bukhdruker, Daria A. Dmitrieva, Dmitry V. Gulnov, Elena V. Nemtseva, Valentin I. Gordeliy, Alexey V. Mishin, Valentin I. Borshchevskiy, Eugene S. Vysotski

**Affiliations:** 1 Photobiology Laboratory, Institute of Biophysics SB RAS, Federal Research Center “Krasnoyarsk Science Center SB RAS”, Krasnoyarsk, Russia; 2 Institute of Fundamental Biology and Biotechnology, Siberian Federal University, Krasnoyarsk, Russia; 3 Research Center for Molecular Mechanisms of Aging and Age-Related Diseases, Moscow Institute of Physics and Technology, Dolgoprudny, Russia; 4 Institut de Biologie Structurale (IBS), Université de Grenoble Alpes, CEA, CNRS, Grenoble, France; 5 Institute of Crystallography, University of Aachen (RWTH), Aachen, Germany; 6 Joint Institute for Nuclear Research, Dubna, Russia

**Keywords:** Biochemistry, Biophysics, Structural biology

## Abstract

Coelenterazine-*v* (CTZ-*v*), a synthetic vinylene-bridged π-extended derivative, is able to significantly alter bioluminescence spectra of different CTZ-dependent luciferases and photoproteins by shifting them towards longer wavelengths. However, Ca^2+^-regulated photoproteins activated with CTZ-*v* display very low bioluminescence activities that hampers its usage as a substrate of photoprotein bioluminescence. Here, we report the crystal structure of semi-synthetic Ca^2+^-discharged obelin-*v* bound with the reaction product determined at 2.1 Å resolution*.* Comparison of the crystal structure of Ca^2+^-discharged obelin-*v* with those of other obelins before and after bioluminescence reaction reveals no considerable changes in the overall structure. However, the drastic changes in CTZ-binding cavity are observed owing to the completely different reaction product, coelenteramine-*v* (CTM-*v*). Since CTM-*v* is certainly the main product of obelin-*v* bioluminescence and is considered to be a product of the “dark” pathway of dioxetanone intermediate decomposition, it explains the low bioluminescence activity of obelin and apparently of other photoproteins with CTZ-*v*.

## Introduction

Bioluminescence is a natural phenomenon of cold light emission from the living organisms, brought into effect by the chemical reaction in which the enzyme, luciferase, catalyzes oxidation of the substrate, luciferin, by the molecular oxygen. There are more than 30 different bioluminescence systems known comprising various luciferins and luciferases^1^. Some of these systems also involve an accessory protein to store a luciferin and deliver it to the luciferase e.g., coelenterazine-binding protein from sea pansy *Renilla*, or an antenna protein to alter the color and quantum yield of a bioluminescence reaction via Förster resonance energy transfer (FRET) mechanism such as famous green fluorescent protein (GFP)^2^.

**Figure 1:**
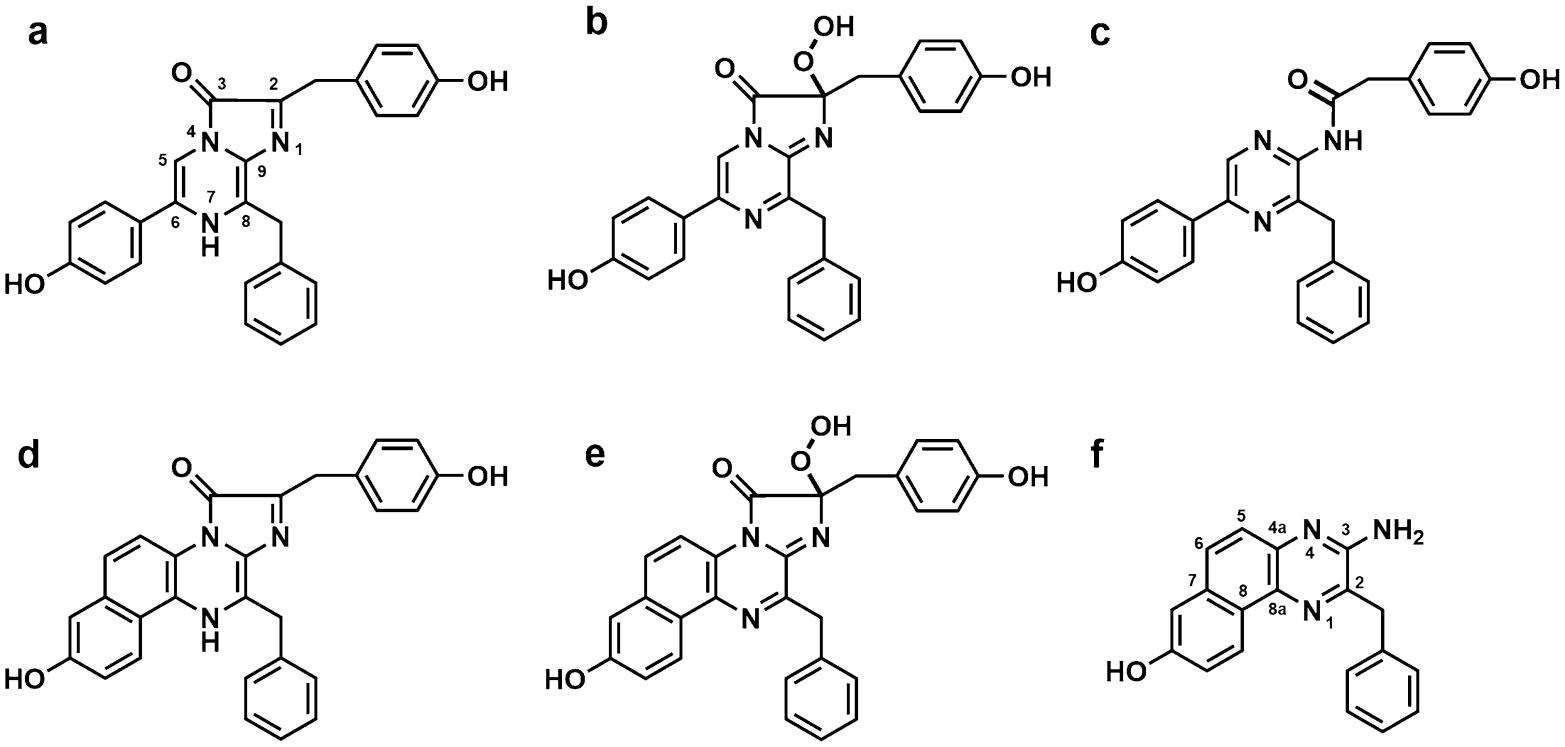
Coelenterazine, coelenterazine-*v* and their derivatives. CTZ (**a**), 2-hydroperoxyCTZ (**b**), CTD (**c**), CTZ-*v* (**d**), 2-hydroperoxyCTZ-*v* (**e**), and CTM-*v* (**f**).

One of the most widespread and well-studied luciferins is coelenterazine (CTZ, Fig. 1a), found to be a substrate of bioluminescent reaction in at least nine phyla of marine luminous organisms including jellyfishes, hydroids, ctenophores, copepods, soft corals, worms, crustaceans, mollusks and vertebrates^1^. In order to oxidize CTZ and produce light, different marine organisms use different enzymes that fall into two categories: classic luciferases such as Renilla, Gaussia, or Metridia ones^3^ and Ca^2+^-regulated photoproteins such as aequorin, obelin, or berovin^1^. In all these cases of various enzymes using CTZ as a luciferin the chemical mechanism of bioluminescence reaction is believed to be the same, however the bioluminescence protein microenvironment plays an important role in shaping the final outcome of each particular reaction thereby offering a wide range of characteristics, such as quantum yield of the reaction, bioluminescence color and kinetics.

Ca^2+^-regulated photoproteins are members of the EF-hand Ca^2+^-binding protein superfamily that provide bright flash-type bioluminescence to a great number of marine cnidarians and ctenophores. Photoproteins consist of a single polypeptide chain with a molecular weight of around 22 kDa to which the oxygenated CTZ, 2-hydroperoxycoelenterazine (2-hydroperoxyCTZ, Fig. 1b), is tightly but non-covalently bound. Photoprotein bioluminescence is greatly accelerated by binding of calcium ions to the EF-hand Ca^2+^-binding sites of a protein. This event, followed by small structural changes within the substrate-binding cavity, initiates rapid decarboxylation of 2-hydroperoxyCTZ with the elimination of CO_2_ and generation of the product, coelenteramide (CTD, Fig. 1c), in an electronically excited state^4^. When the excited CTD relaxes to the ground state, light emission occurs with λ_max_ in the range of 465–495 nm depending on the photoprotein type.

As for now, the CTZ-dependent bioluminescence proteins have been successfully applied to the intracellular calcium measurements, monitoring of biological processes such as gene expression, protein–protein interactions, and disease progression, as biosensors in immunoassays, nucleic acid hybridization assays and in vivo imaging^5^. Brought to life by the prospects of various analytical applications of CTZ-dependent bioluminescence proteins, the approach of chemical modification of CTZ to acquire luciferins which would alter properties of these proteins became very popular. In order to change bioluminescence intensity, kinetics, calcium sensitivity, or generate different emission color, a large number of CTZ analogues with various modifications of imidazopyrazinone core or its C2, C6 and C8 substituents have been synthesized and tested in many applications with different photoproteins or luciferases showing promising results^6–10^.

These endeavors were mainly aimed to shift light emission to the longer wavelength region with a view of increasing the detection sensitivity of CTZ-dependent bioluminescent proteins in deep mammalian tissues at their use as reporters for in vivo assays. Since light has its maximum depth of penetration into tissues in the near-infrared window, the demand for the reporters emitting red light is always high. Among the most promising CTZ analogues is coelenterazine-*v* (CTZ-*v*, Fig. 1d), a vinylene-bridged π-extended derivative, that significantly shifts bioluminescence spectra of different CTZ-dependent luciferases and photoproteins when used as a reaction substrate^11^. Among these proteins are native Renilla and Oplophorus luciferases, whose bioluminescence spectra are shifted from 475 to 512 nm and from 454 to 480 nm, respectively^12^. Moreover, certain luciferase mutants display even greater spectral shifts with CTZ-*v,* which makes it even more encouraging to consider CTZ-*v* as a powerful substrate for in vivo imaging. Using mutants of Renilla luciferase with bioluminescence maxima at 547 nm in combination with CTZ-*v* as a luciferin, it was possible to shift bioluminescence emission up to λ_max_ = 588 nm^13^. CTZ-*v* has the same effect on photoprotein bioluminescence spectra; obelin activated with CTZ-*v* (obelin-*v*) demonstrates red-shifted bioluminescence with a peak at 532 and a shoulder at 415 nm while obelin with native CTZ emits light with a maximum at 480 nm and a shoulder at 400 nm^14,15^.

However, it quickly became clear that CTZ-*v* affects not only spectral properties of the bioluminescence proteins but also the bioluminescence activity and the reaction quantum yield, with the outcome varying from protein to protein. In the case of Renilla luciferase, for example, the use of CTZ-*v* significantly increases the peak light intensity, providing at that the total light yield comparable to the one of the native CTZ (73%)^12^. On the contrary, the light outputs of Ca^2+^-regulated photoproteins and Oplophorus luciferase with CTZ-*v* are drastically reduced. Thus, when activated by CTZ-*v*, aequorin and obelin retain only 3.5% and 1.7%, correspondingly^12,14^, of the total light yield of photoprotein activated with unmodified CTZ. Unfortunately, it hinders the usage of CTZ-*v* analogue as a substrate of the photoprotein bioluminescence.

To shed light on the reasons behind the low efficiency of photoprotein bioluminescence with CTZ-*v,* the crystal structure of Ca^2+^-regulated photoprotein obelin bound with 2-hydroperoxy adduct of CTZ-*v* (obelin-*v*) at 1.80 Å resolution was recently solved^15^. This structure provided useful information about coelenterazine-binding cavity of obelin-*v* before the bioluminescence reaction. Unexpectedly, the structures of obelin-*v* and obelin bound with native CTZ revealed almost no difference; only minor rearrangement in hydrogen-bond pattern and slightly increased distances between the key active site residues and 2-hydroperoxyCTZ-*v* were found. Thus, the minor changes in the substrate microenvironment observed in the obelin-*v* active site were proposed to account for the low efficiency of generating an electronic excited state (*Φ*_*S*_) resulting in a low bioluminescence activity of obelin-*v*^15^.

To deepen our understanding of the CTZ-*v* behavior as a luciferin of photoprotein bioluminescence, determination of the crystal structure of Ca^2+^-discharged obelin-*v* was also required, i.e., the structure of obelin-*v* after bioluminescence reaction with the product bound within the protein active site. In this paper we present the crystal structure of Ca^2+^-discharged obelin-*v* bound with the product of bioluminescence reaction at 2.1 Å resolution as well as time-resolved fluorescence properties of Ca^2+^-discharged obelin-*v*.

## Results and discussion

### Overall structure of Ca^2+^-discharged obelin-v and the structure of the reaction product

**Figure 2:**
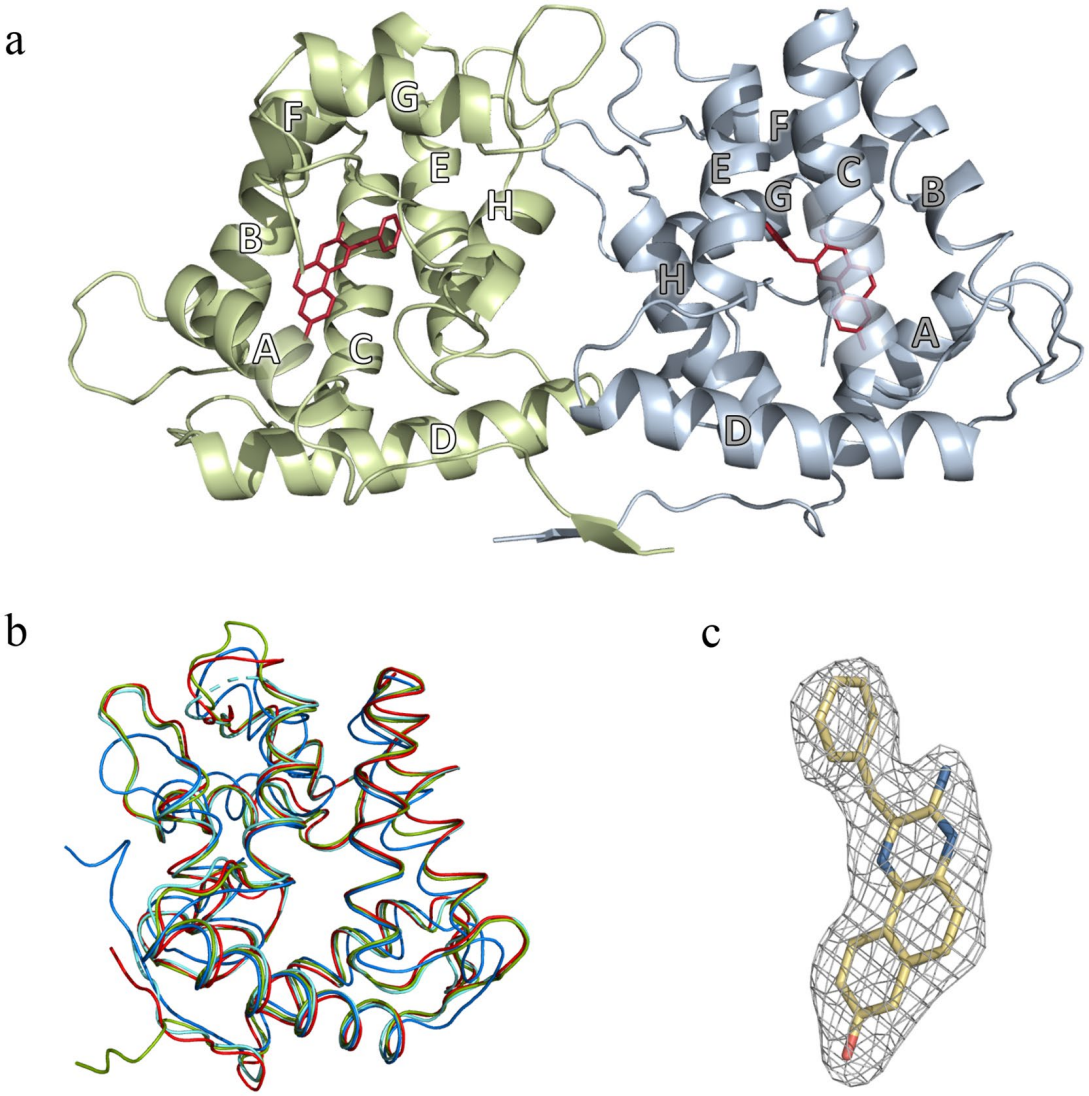
Crystal structure of Ca^2+^-discharged obelin-*v*. (**a**) Overall structure of Ca^2+^-discharged obelin-*v*. The helices are marked by capital letters A–H. (**b**) Superimposition of Ca^2+^-discharged obelin-*v* (green, PDB: 8A9S), Ca^2+^-discharged obelin W92F with no Ca^2+^ (cyan, PDB: 1S36), Ca^2+^-discharged obelin bound with three Ca^2+^ ions (blue, PDB: 2F8P) and obelin-*v* (red, PDB: 7O3U). (**c**) 2F_o_–F_c_ electron density map of CTM-*v* molecule in the substrate-binding cavity of Ca^2+^-discharged obelin-*v* (chain A) countered at 1σ.

The crystal structure of Ca^2+^-discharged obelin-*v* contains two protein molecules per asymmetric unit (Fig. 2a). The final model includes two protein chains (A, B) with 192 and 191 of the 195 amino acid residues, correspondingly, two coelenteramine-*v* (CTM-*v*) molecules, six DMSO molecules, and 341 solvent molecules. The residues 1–3 in protein chain A and 1–4 in chain B are not visible in the electron-density maps, as is generally observed for the *N*-terminal residues in the structures of other Ca^2+^-regulated photoproteins.

Due to the crystallization conditions Ca^2+^-discharged obelin-*v* structure does not contain bound Ca^2+^ ions, because citrate is a calcium chelator. This makes Ca^2+^-discharged obelin-*v* the second photoprotein spatial structure obtained in this particular conformation, i.e., with the reaction product but without Ca^2+^ ions, the first one being the Ca^2+^-discharged W92F obelin structure at 1.96 Å resolution (PDB: 1S36)^16^.

However, in the first Ca^2+^-binding loop of Ca^2+^-discharged obelin-*v* in both protein chains A and B (residues 29–37) there is an electron density which plausibly corresponds to Na^+^. The ion is coordinated by Asp30, Asn32, Asn34, Lys36 and two water molecules with an average of ~ 2.4 Å separation to oxygen atoms that is typical for coordination of both Ca^2+^ and Na^+^^17^. At the same time, Na^+^ can be straightforwardly distinguished from Ca^2+^ by electron density, B factors and better R_free_ values during refinement (lower by 0.7% in the case of Na^+^)^17^. Noteworthy is that the crystals of Ca^2+^-discharged W92F obelin were also obtained using sodium citrate and Na^+^ is also found in the first Ca^2+^-binding site^16^.

The structures of chains A and B are almost identical except for the orientations of some side chains on the surface and labile regions of loops and *N*-terminus. The RMSD of Cα atoms of chain A vs chain B is only 0.22 Å. The notable distinctions were observed in the loop formed by the residues 122–131 which is a part of the EF-hand Ca^2+^-binding site III that apparently is due to the contacts between the two chains of the dimer. There is a salt bridge defined between Asp123 of the chain A and Asn162 of the chain B, while there is no contact between Asp123 of the chain B and Asp162 of the chain A. Additionally, the conformation of 122–131 loop could be influenced by the presence of intramolecular interaction between Lys124 and Asp134 in the chain A and its absence in the chain B.

Ca^2+^-discharged conformation of obelin-*v* is a globular molecule with the radius of ~ 25 Å, formed by the two sets of four helices designated as A–D and E–H in the *N*- and *C*-terminal domains, respectively (Fig. 2b). Its overall structure strongly resembles those of both obelins bound with 2-hydroperoxy adducts of native CTZ (PDB: 1QV0) and CTZ-*v* (PDB: 7O3U) before the bioluminescence reaction^15,18,19^, and that of Ca^2+^-discharged conformation of obelin with native CTD after the reaction (PDB: 2F8P)^20^. For example, RMSD of Cα atoms of obelin-*v* bound with 2-hydroperoxyCTZ-*v* (Fig. 1e) before the reaction (PDB: 7O3U) vs chain A of the Ca^2+^-discharged obelin-*v* after the reaction is only 0.68 Å (Fig. 2b).

The most interesting feature of Ca^2+^-discharged obelin-*v* structure is that the reaction product in its CTZ-binding cavity turned out to differ from CTD (Fig. 1c), which is so far observed in all solved structures of Ca^2+^-discharged photoproteins, including obelin (PDB: 2F8P) and its various mutants, such as F88Y (PDB: 4N1G), W92F (PDB: 1S36), and Y138F (PDB: 4MRY)^16,20–22^. The electron density distribution in the active site of Ca^2+^-discharged obelin-*v* strongly suggests the presence of *v*-modified coelenteramine (CTM-*v*) (Figs. 1f, 2c).

The formation of CTM from CTZ was previously reported by Shimomura in his attempts to identify the light-emitting moiety of natural aequorin using denaturing conditions^23^. The substance isolated at that time was described as most likely 2-amino-3-benzyl-5-(*p*-hydroxyphenyl)pyrazine, i.e., CTM. Later, CTD was shown to be the main product of the aequorin bioluminescence reaction, however, HPLC analysis revealed the presence of CTM^24^, indicating the established pathway for CTM generation during bioluminescence.

**Figure 3:**
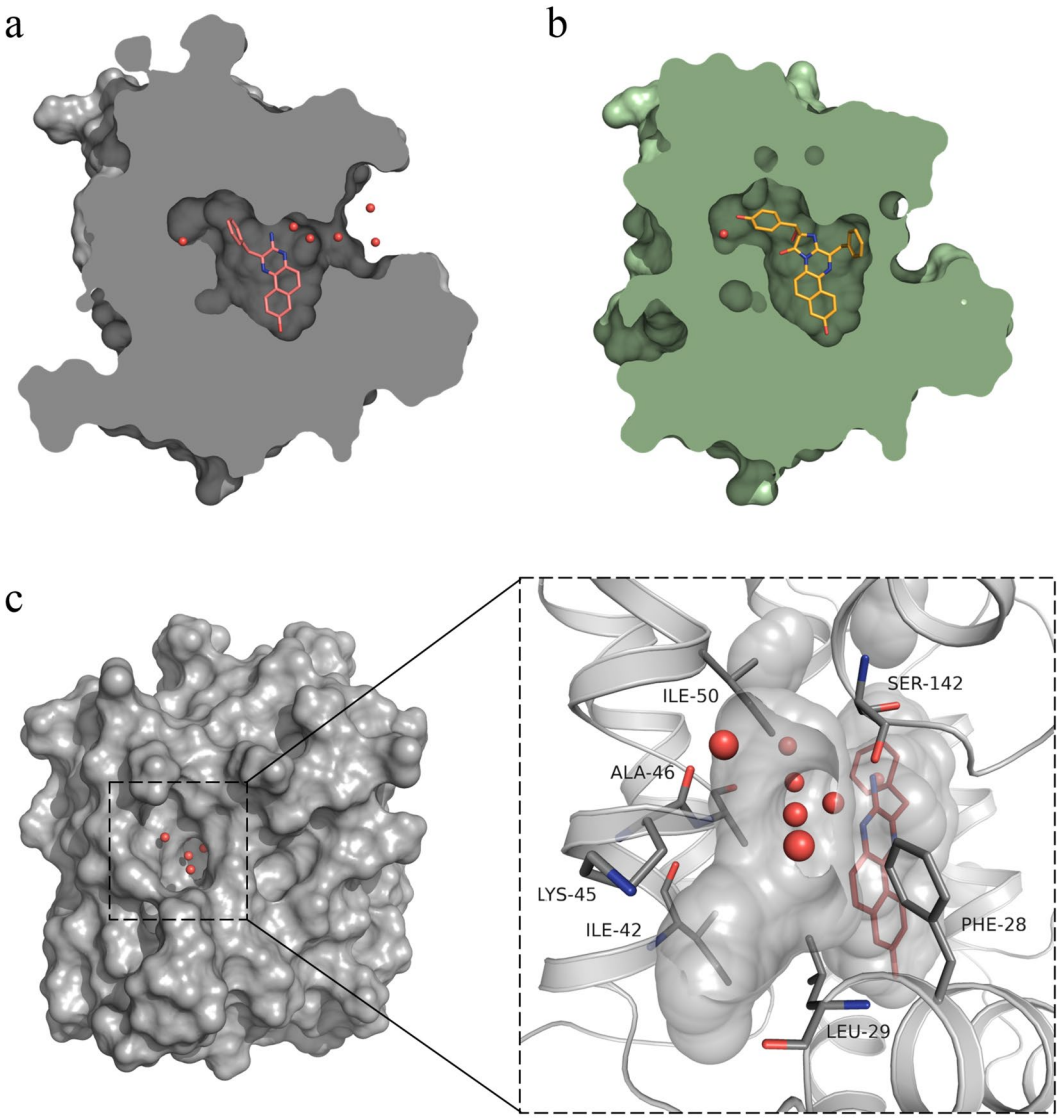
Molecular-surface representation of obelin-*v*. CTM-*v* (**a**) and 2-hydroperoxyCTZ-*v* (**b**) molecules within the substrate-binding cavities of the corresponding obelin-*v* structures: Ca^2+^-discharged obelin-*v* (gray, PDB: 8A9S) and active obelin-*v* (green, PDB: 7O3U). CTM-*v* and 2-hydroperoxyCTZ-*v* molecules are colored in red and yellow, respectively. Both structures are in the same orientation. (**c**) Ca^2+^-discharged obelin-*v* (8A9S, chain B) with a solvent-exposed opening on the surface shown in the center. ((**c**), insert) Detailed representation of the solvent-exposed opening and the ligand-binding pocket with the CTM-*v* and water molecules. The CTM-*v* and amino acid residues surrounding the solvent-exposed opening are respectively shown as stick models of red and grey colors. Water molecules are represented as red balls.

CTM-*v* resides in hydrophobic internal cavity of Ca^2+^-discharged obelin in the same position but in different orientation compared to 2-hydroperoxyCTZ-*v* of obelin-*v* (Fig. 3a,b). CTM-*v* is rotated 180 degrees, so that 2-benzyl group of CTM-*v* is directed towards different set of amino acid residues compared to the corresponding 8-benzyl group of 2-hydroperoxyCTZ-*v*. Of note is that there is not enough space for the ligand to rotate this way in current conformation. The rotation of the substrate could happen either during Ca^2+^-induced bioluminescence reaction when 2-hydroxyphenyl group is removed from the substrate molecule and subsequently CTM-*v* is formed*,* or after bioluminescence reaction is ceased and calcium ions left the protein owing to crystallization conditions. When calcium ions leave Ca^2+^-binding sites of Ca^2+^-discharged obelin-*v*, the protein definitely undergoes conformational changes that might provide enough space in the CTZ-binding pocket for the substrate rotation in question.

The RMSD Cα of chain A of Ca^2+^-discharged obelin-*v* vs Ca^2+^-discharged W92F obelin (PDB: 1S36) is only 0.51 Å (Fig. 2b). However, there are some regions displaying notable differences between the structures. In Ca^2+^-discharged obelin-*v* there is a 2.9 Å shift of helix B towards the center of the protein molecule around residues 45–50 (if measured at Ala46 Cα). In Ca^2+^-discharged W92F obelin the benzyl group of CTD is oriented directly to helix B and pushes it out a bit. At the same time the CTM-*v* of Ca^2+^-discharged obelin-*v* is located deeper inside the active center due to the smaller size and its benzyl group is oriented towards helix E. The similar 3.2 Å shift of the helix B (if measured at Ala46 Cα) is present between active obelin-*v* (PDB: 7O3U) and Ca^2+^-discharged obelin-*v* structures that is also related to the benzyl moiety of 2-hydroperoxyCTZ-*v* pushing the helix B out. Thus, these changes are certainly conditioned by the different size of the reaction product molecule bound in the active center, since CTM-*v* is much smaller than 2-hydroperoxyCTZ-*v* and CTD-*v*.

In both obelin and obelin-*v* containing the corresponding 2-hydroperoxyCTZs, the *C*-terminus effectively caps the substrate-binding cavity, ensuring a solvent-inaccessible and nonpolar environment of the CTZ derivative^15,19^. The inaccessibility of the internal substrate-binding cavity of photoproteins to solvent is additionally guarded by the hydrogen bonds formed by Arg21 located in helix A and *C*-terminal Pro as well as Asp187 and Phe178^25,26^.

After the bioluminescence reaction Ca^2+^-discharged obelin-*v* still retains the above mentioned organization of *C*-terminus cap. However, Ca^2+^-discharged obelin-*v* also has a possible solvent-access opening located between Phe28, Leu29 of helix A, Ile42, Ala46, Ile50 of helix B, and Ser142 of helix F (Fig. 3c). Relative to the structure of obelin-*v* before bioluminescent reaction (PDB: 7O3U), repositioning of Lys45 and Asp49 and breaking of a hydrogen bond between Lys45 and Ser142 can be detected simultaneously with the solvent-access opening appearance. This might be attributed to the high flexibility of Lys45, which could be positioned in four different orientations (two per each chain) and two of which provide a surface opening in question. It is worth mentioning that this ‘hole’ is commonly observed in the structures of other Ca^2+^-discharged photoproteins, for example, obelin with both CTD and calcium ions or Y138F obelin (PDB codes: 2F8P, 4MRY)^20,22^.

### Hydrogen bond network in the substrate-binding cavity of Ca^2+^-discharged obelin-v

**Figure 4:**
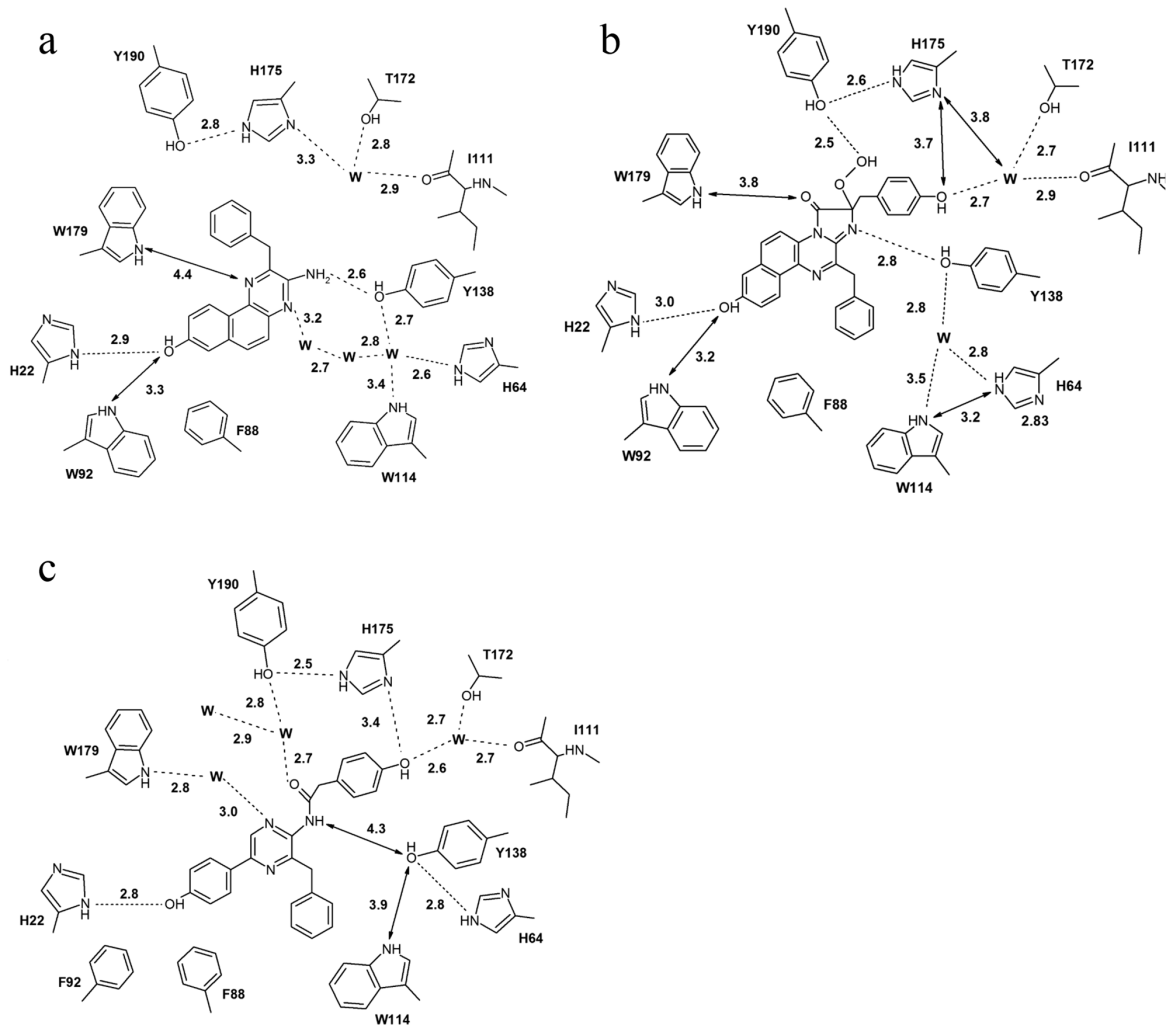
Two-dimensional representation of the hydrogen bond network. (**a**) Ca^2+^-discharged obelin-*v* (PDB: 8A9S, chain A), (**b**) active obelin-*v* (PDB: 7O3U) and (**c**) Ca^2+^-discharged W92F obelin (PDB: 1S36). Hydrogen bonds are shown as dashed lines, distances between atoms are shown as arrows, ‘w’ stands for a water molecule. Distances are given in Å.

Despite minor overall changes when compared to other obelin structures, the organization of substrate-binding cavity of Ca^2+^-discharged obelin-*v* undergoes drastic readjustment. Amino acid residues facing the CTM-binding cavity of Ca^2+^-discharged obelin-*v* generally retain the same position and orientation contrary to obelin-*v* and Ca^2+^-discharged W92F structures (PDB: 7O3U and 1S36, respectively) (Fig. 4). However, a new ligand with its different orientation results in the formation of a new hydrogen bond network as CTM-*v* is rotated ~ 180 degrees relative to 2-hydroperoxyCTZ-*v* in active obelin-*v* and CTD in Ca^2+^-discharged W92F structures (PDB: PDB: 7O3U an 1S36, respectively).

Despite the ligand rotation, the OH group of CTM-*v* forms a hydrogen bond with His22*,* which is the bond commonly observed in other photoprotein structures (Fig. 4). The second hydrogen bond formed by CTM-*v* directly with the adjacent amino acid residues is the bond between Tyr138 and amino group of CTM-*v*. This hydrogen bond is unique for the CTM-*v*; in both obelin and obelin-*v*, Tyr138 is hydrogen-bonded with N1 of both 2-hydroperoxyCTZs and moves away from CTD at the distance of 4.3 Å in Ca^2+^-discharged W92F obelin (Fig. 4). Additionally, CTM-*v* is hydrogen-bonded with the water molecule via its N4 nitrogen atom.

The CTZ-binding cavity of Ca^2+^-discharged obelin-*v* contains several water molecules (Fig. 4a). In chain A one water molecule is hydrogen-bonded with Ile111, Thr172 and His175, the three others are located near His64, Tyr138 and Trp114 cluster. One of the molecules is at the hydrogen bond distance from Tyr138, His64 and Trp114, the other is hydrogen-bonded to N4 atom of CTM-*v*, and the third one resides between these two water molecules (Fig. 4a). In chain B the organization of the hydrogen bond network is generally the same with only slight differences in the distances of the above mentioned hydrogen bonds.

In the available obelin structures a number of water molecules located in the substrate-binding pocket and their distribution are different. In active obelin-*v* there are two water molecules—one of them is hydrogen-bonded to Ile111 backbone, Thr172 and the OH group of 2-(*p*-hydroxy)-benzyl substituent of 2-hydroperoxyCTZ-*v* and another molecule is stabilized by the hydrogen bonds with Tyr138, His64 and Trp114 (Fig. 4b). In Ca^2+^-discharged W92F obelin one water molecule forms hydrogen bonds with hydroxybenzyl group of CTD, Ile111 backbone and Thr172, while the other water molecules are located near His175, Trp179 and Tyr190 triad, forming hydrogen bonds with N3 and carbonyl O atom of CTD (Fig. 4c). Obviously, it is the solvent-access opening that allows water molecules to diffuse into the cavity from the surface and to fill the free space (Fig. 3c), which appeared in the CTZ-binding pocket because of the smaller size of CTM-*v* in comparison with 2-hydroperoxyCTZ and CTD.

### Time-resolved fluorescence of Ca^2+^-discharged obelin-v

The finding that CTM-*v* is the main product of obelin-*v* bioluminescence raises the question of the effect of its photophysical characteristics on the efficiency of the bioluminescent reaction. Generally, Ca^2+^-regulated photoproteins with peroxy adducts in the active sites are non-fluorescent, but after the bioluminescence reaction ceases, they become capable of fluorescing in the visible spectral range for as long as the reaction product remains bound within a substrate-binding cavity of the photoprotein. Earlier we demonstrated that the fluorescence spectrum of Ca^2+^-discharged obelin-*v* differs significantly from that of obelin: under excitation at 373 nm, the two peaks about 530 nm and 435 nm emerge, while obelin emission has maximum around 515 nm and a weak shoulder at 415 nm^15^.

**Figure 5:**
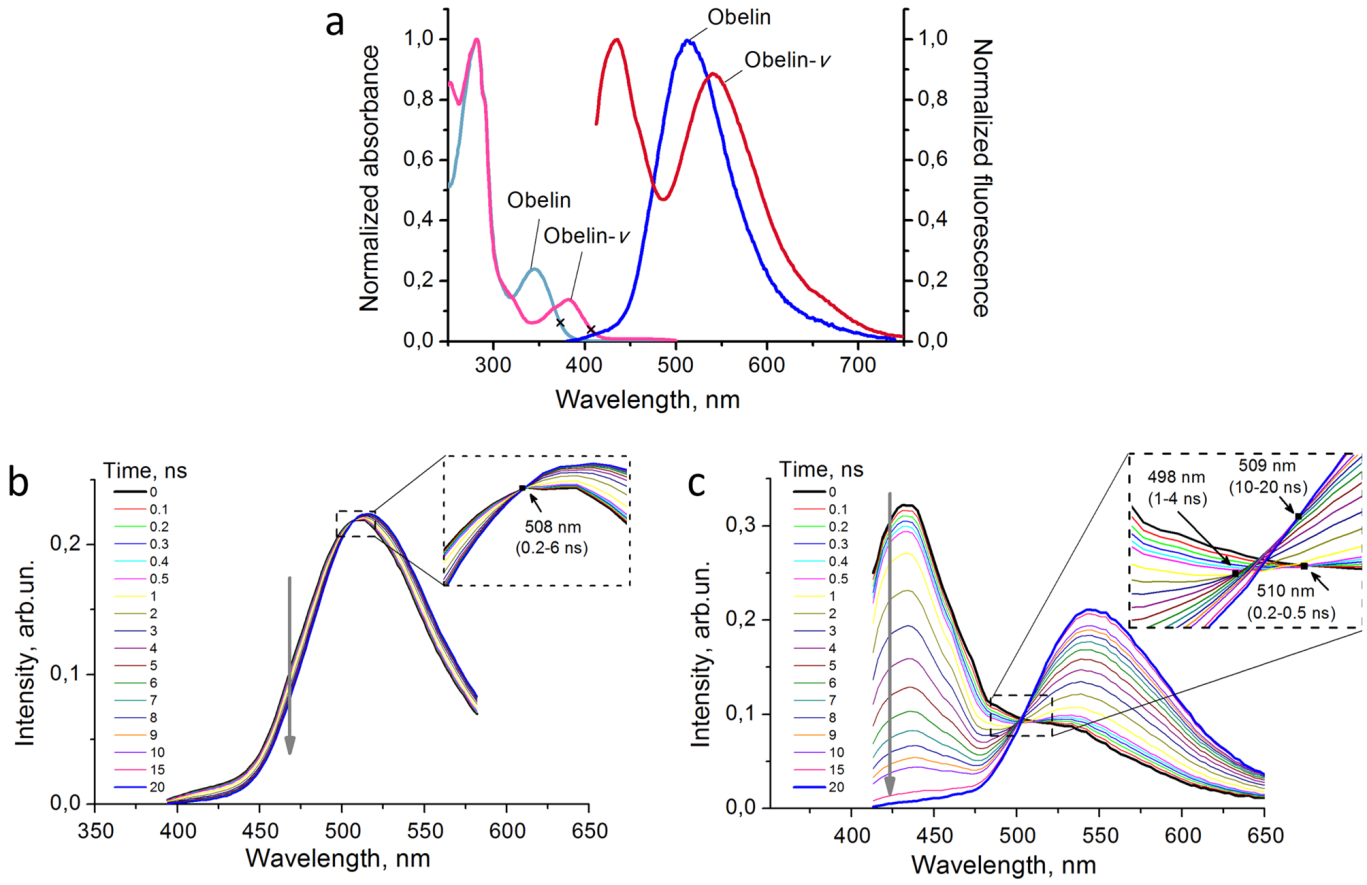
Fluorescence properties of Ca^2+^-discharged obelin and obelin-*v*. (**a**) Absorption (left) and fluorescence (right) spectra of Ca^2+^-discharged obelin (cyan and blue colors) and obelin-*v* (pink and red colors). Spectra are normalized to the maximum. Fluorescence excitation wavelength was 373 nm for obelin and 407 nm for obelin-*v* (shown as crosses on the spectra). (**b**) TRANES of Ca^2+^-discharged obelin, excitation at 373 nm and (**c**) Ca^2+^-discharged obelin-*v*, excitation at 407 nm. Inserts show the area of isoemissive points which are indicated with markers. Gray arrows show a direction of the bands change in time.

Time-resolved fluorescence spectroscopy, which allows estimating basic photophysical properties and quantity of the emitters contributing to the spectra is a more sensitive technique for studying complex fluorescence spectra. Since the absorption band of Ca^2+^-discharged obelin-*v* is red-shifted by > 30 nm relative to that of the Ca^2+^-discharged obelin, two different excitation wavelengths at the red edge of the corresponding spectra (407 nm and 373 nm for Ca^2+^-discharged obelin-*v* and obelin, respectively) have been used to measure their steady-state and time-resolved fluorescence (Fig. 5a).

Time-resolved fluorescence decays of Ca^2+^-discharged obelin and obelin-*v* have been measured across the emission spectra with a step of 2 nm and 3 nm, respectively. Decay sets were processed with two approaches: by constructing either time-resolved area-normalized emission spectra (TRANES) or decay-associated spectra (DAS). The first method aims at counting a number of fluorophores providing transformation of the emission spectrum in a nanosecond scale^27^. The second approach allows distinguishing spectral distribution of the emitters with different fluorescence lifetimes within experimentally observed total spectrum^28^.

For Ca^2+^-discharged obelins, the time-resolved area-normalized emission spectra are shown in Fig. 5. In the TRANES we identified one isoemissive point for Ca^2+^-discharged obelin and three—for Ca^2+^-discharged obelin-*v* that indicates the presence of two and four emitters with distinct fluorescence lifetimes, respectively^29^. The presence of two fluorophores in obelin spectra could not be surprising, since, along with the intense band peaked at 515 nm, which is usually attributed to the anionic form of CTD, there is also a weak shoulder around 415 nm (Fig. 5a). This additional band is usually referred to a neutral form of CTD. However, a position of the isoemmisive point in the obelin TRANES (508 nm) is far from the spectral range of the neutral form (Fig. 5b). Moreover, earlier the fluorescence lifetime of neutral CTD was estimated as ~ 45 ps^30^, therefore, its kinetics could hardly contribute to the spectral changes at 6 ns, as we observed (Fig. 5b). Thus, besides the main fluorophore, TRANES of Ca^2+^-discharged obelin indicate the presence of some additional emitter, which spectrally and kinetically differs from the neutral form of CTD.

The fluorescence spectrum of Ca^2+^-discharged obelin-*v* is much more complex (Fig. 5a). Under excitation at 407 nm we observed redistribution of intensity between the two peaks relative to fluorescence under excitation at 373 nm^15^, which suggest the presence of multiple fluorophores with different absorption spectra (Fig. S1). TRANES method revealed the presence of at least four emitters within two distinct bands of the Ca^2+^-discharged obelin-*v* fluorescence (Fig. 5c). During TRANES change, only the left spectral peak decreased in emission intensity. Therefore, since three isoemissive points were identified we assume the decay of three emitters in the left band, while most of the right band’s fluorescence originates from a single long-living emitter.

In order to reveal photophysical characteristics of the emitters found, we obtained DAS of both Ca^2+^-discharged obelins. For this, the model of discrete lifetimes and the method of global analysis were applied to the fluorescent decay sets. DAS characteristics are presented in Table 1. It should be mentioned that the model of discrete lifetime components allowed good fitting of decays throughout the emission spectra of the obelins (statistical criterion χ^2^was close to 1). For Ca^2+^-discharged obelin, two lifetime components assured satisfactory fitting quality, while obelin-*v* dataset required four lifetime components.

**Table 1:** Fluorescence lifetime components of Ca^2+^-discharged obelin and obelin-*v* fluorescence.

Ca^2+^-discharged protein	Spectral range/excitation wavelength	τ_i_, ns	*f*_i_, %	λ_i_^max^, nm	χ^2^
Obelin	394–582 nm/373 nm	1.52	12.4	482	0.99
		4.65	87.6	512	
Obelin-*v*	413–650 nm/407 nm	0.27	5.1	440	1.04
		1.62	26.4	434	
		3.23	23.1	440	
		8.37	45.4	548	

τ_i_—lifetime component calculated by global analysis of the decays within indicated spectral range; *f*_i_—spectral fraction of the component with τ_i_ in total emission spectrum; λ_i_^max^—spectrum maximum, associated with τ_i_, (DAS_i_); χ^2^—statistical criterion characterizing the quality of analysis using indicated lifetime components and their amplitudes.

It was found that the above mentioned additional emitter in the Ca^2+^-discharged obelin sample has the fluorescence lifetime of about 1.52 ns and the spectrum peaked near 480 nm (Table 1). Its spectral contribution is low (12.4%) as compared to the main emitter with a lifetime of 4.65 ns. This is the reason we observed small overall TRANES change for this photoprotein (Fig. 5b). For Ca^2+^-discharged obelin-*v*, three different lifetime components were obtained (0.27, 1.62 and 3.23 ns) with very similar spectral properties peaked at 434–440 nm. This spectral similarity was to be expected, because the positions of TRANES isoemissive points were close to each other (Fig. 5c). The emission of the right band with a maximum around 540 nm is probably provided mostly by one fluorophore with lifetime of about 8.4 ns. At the moment, we can only assume the identity of the found emitters, since the detailed fluorescent properties of the different ionic forms of CTM, CTD, and even more so of CTM-*v* and CTD-*v*, have not yet been studied.

We also analyzed the time-resolved properties of the Ca^2+^-discharged obelin-*v* under excitation at 373 nm and found out that fluorescence lifetime values closely approximated those under excitation at 407 nm, whereas their contributions slightly differed compared to the parameters obtained under excitation at 407 nm (Table S1). This was expected, since the steady-state fluorescence spectrum of Ca^2+^-discharged obelin-*v* does depend on the excitation wavelength (Fig. S1), indicating the presence of multiple fluorophores in the sample.

In this study, the crystal structure of Ca^2+^-discharged obelin-*v* revealed CTM-*v* to be the predominant product of the photoprotein bioluminescence reaction. It is not the first time, however, when CTM was identified as a possible product of this reaction, namely in aequorin, and while the main product was established to be CTD, according to the HPLC analysis the CTM presence was considerable^24^. Recently, the formation of CTM from 2-hydroperoxyCTZ was thoroughly investigated under several conditions including native aequorin bioluminescence reaction and various denaturing treatments^31^. The ratio between the two products of aequorin bioluminescence reaction, namely CTD and CTM, was estimated to be 100:13.5 and two possible pathways of CTM generation from 2-hydroperoxyCTZ were proposed. This ratio explains why previously X-ray protein crystallography has never shown CTM as a product in any Ca^2+^-discharged photoprotein structure solved as chances to crystallize a protein with a side product bound are generally low.

Since TRANES of Ca^2+^-discharged obelin denote the presence of two distinct emitters (Fig. 5b) and we can rule out the neutral form of CTD due to the spectral mismatch, it is reasonable to assume these two registered components to be other ionic forms of CTD and CTM. It was proved earlier that CTD can exist in different ionic states: a neutral species with fluorescence emission maximum around 400 nm, the amide anion with maximum around 450 nm, the phenolate anion (480–490 nm), and the pyrazine-N(4) anion resonance form (535–550 nm)^32^. Two fluorescence components of the Ca^2+^-discharged obelin identified by DAS approach have spectral maxima around 482 nm and 512 nm (Table 1). The latter one, which is the main component could be attributed to either phenolate anion or pyrazine anion resonance form of CTD, which is supported by the structural data about CTD being the main product of most photoprotein bioluminescence reactions.

The second component with the maximum around 482 nm, however, could be attributed to either phenolate anion of CTD or some ionic form of CTM. Recently, CTM was also found in Ca^2+^-discharged photoprotein berovin from ctenophore *Beroe abyssicola* as a bioluminescence product together with CTD, and according to the RP-HPLC their ratio was 75:100^33^. Fluorescence maxima of both CTD and CTM extracted from Ca^2+^-discharged berovin were found to be 440 nm in 50% acetonitrile.

**Figure 6:**
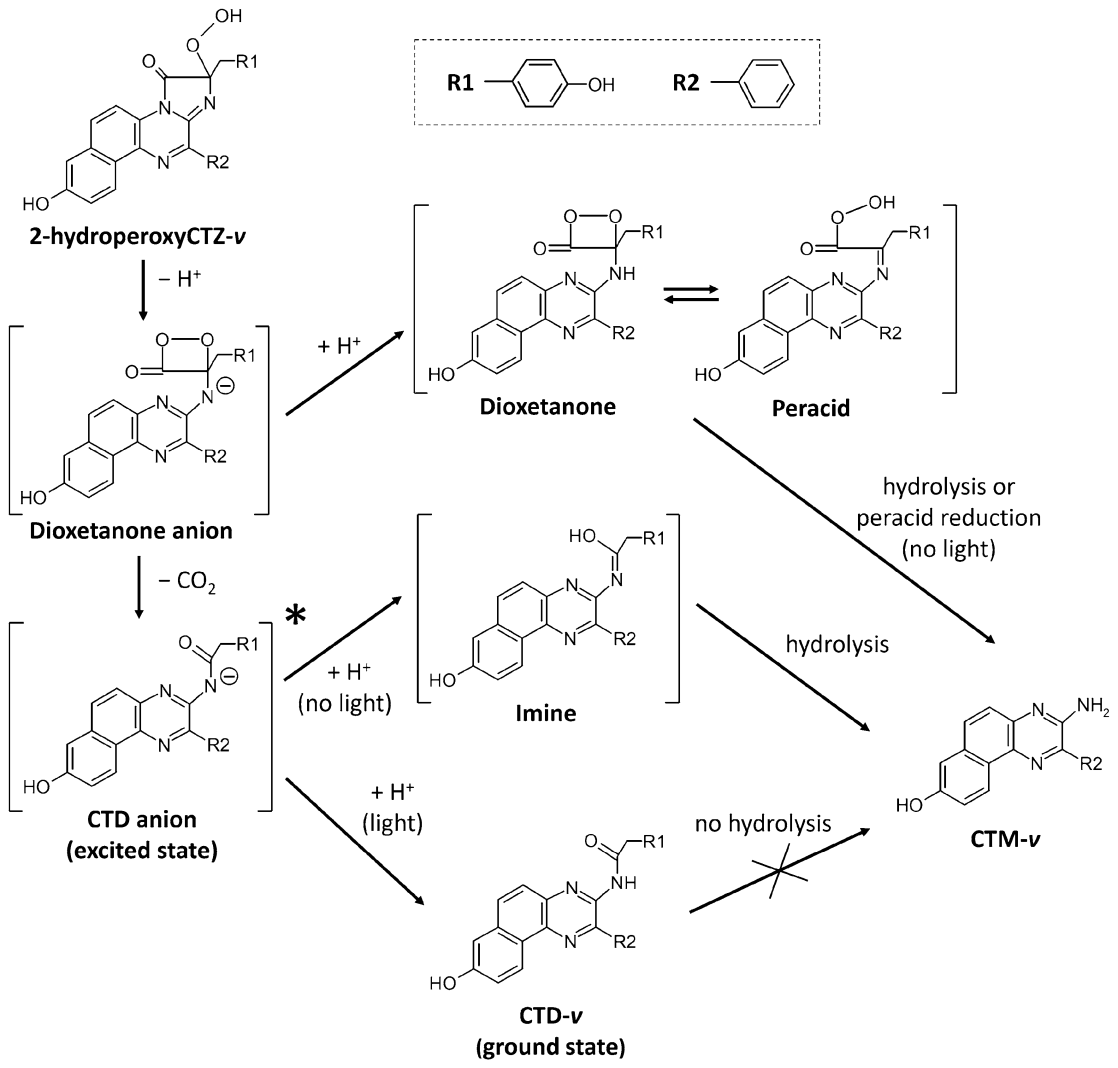
Plausible pathways of CTM-*v* generation in Ca^2+^-dependent bioluminescence reaction of obelin.

Likewise, the four emitters found in the Ca^2+^-discharged obelin-*v* could be attributed to different ionic forms of CTD-*v* and CTM-*v*. Taking into account Ca^2+^-discharged obelin-*v* structural data with CTM-*v* as the main product and the lack of spectral data on CTD-*v* or CTM-*v*, it is hard to speculate on the emitter distribution. However, these data can definitely explain the low bioluminescence activity of obelin-*v*, since CTM is considered to be a product of “dark” pathway of dioxetanone intermediate decomposition of photoprotein bioluminescence reaction (Fig. 6)^31^.

It is interesting to note that according to the proposed pathways of CTM generation in the photoprotein bioluminescence reaction, its formation always occurs without light emission independently on whether CTM is generated as a product of direct dioxetatone hydrolysis (or imine hydrolysis, or peracid reduction) or as a product of CTD anion hydrolysis with imine as an intermediate (Fig. 6). According to the recent study on CTM formation^31^, it is not possible for the CTD to be hydrolyzed to CTM after the excited CTD anion relaxes to the ground state with the emission of light. Thus, if most of the obelin-*v* bioluminescence and fluorescence emitters are different ionic forms of CTM-*v*, then there is only a small amount of CTD-*v* generated, which formation is responsible for 1.7% of bioluminescence activity of obelin-*v* as compared to that of the obelin activated with unmodified CTZ.

## Conclusions

In this study, we report the crystal structure of the semi-synthetic Ca^2+^-discharged obelin bound with CTM-*v* determined at 2.1 Å resolution*.* Comparison of the crystal structure of Ca^2+^-discharged obelin-*v* with that of obelin bound with 2-hydroperoxyCTZ-*v* or with Ca^2+^-discharged obelin with CTD reveals no significant changes in the overall structure. However, drastic changes in CTZ-binding cavity are observed due to the completely different reaction product, CTM-*v*. Time-resolved fluorescence measurements discovered the presence of at least four emitters in Ca^2+^-discharged obelin-*v*, which might be attributed to different ionic forms of CTM-*v* and CTD-*v*. Since CTM-*v* is identified as the main product of obelin-*v* bioluminescence by structural data and can be considered as a product of a “dark” pathway of the decomposition of dioxetanone intermediate, it explains the low bioluminescence activity of obelin-*v* relative to that of obelin activated with unmodified CTZ. This explanation is also supported by the quantum yields of bioluminescence reaction (*Φ*_BL_) of cnidarian and ctenophore Ca^2+^-regulated photoproteins^34,35^. The *Φ*_BL_ value of aequorin bioluminescence amounts to 0.19, while *Φ*_BL_ of berovin is more than twice lower (0.083). This difference correlates well with the amount of CTD and CTM isolated from Ca^2+^-discharged aequorin and berovin after bioluminescence reaction^31,33^.

## Methods

### Materials

Native CTZ and CTZ-*v* were obtained from NanoLight Technology, a division of Prolume Ltd. (Pinetop, AZ, USA). Other chemicals, unless otherwise stated, were from Sigma–Aldrich and were the purest grade available.

### Protein preparation

For apophotoprotein production, *Escherichia coli* BL21-Gold (DE3) Codon Plus (RIPL) cells transformed with pET19-OL8 plasmid^36^ carrying the cDNA encoding wild type obelin from *Obelia longissima*^37,38^ were cultivated with vigorous shaking at 37 °C in LB medium containing ampicillin (200 µg/ml). Protein expression was induced with 0.5 mM IPTG at OD_590_ 0.6–0.8 and the cultivation was continued for another 3 h. Recombinant apo-obelin was extracted from *E. coli* inclusion bodies by 6 M urea as previously reported^39^. Apo-obelin was purified on a DEAE-Sepharose Fast Flow column (GE Healthcare, USA), and then concentrated with the use of 10 kDa Amicon Ultra Centrifugal Filters (Merck Millipore, USA). The concentration of apo-obelin was determined using the corresponding molar extinction coefficient at 280 nm calculated with the ProtParam tool^40^.

To produce active photoproteins, apo-obelin was incubated overnight with a twofold molar excess of CTZ or CTZ-*v* in a buffer 5 mM EDTA, 10 mM DTT, 20 mM Tris–HCl pH 7.2 at 4 °C. Active photoproteins were separated from apo-obelin, unbound CTZs, and DTT by ion-exchange chromatography on HiTrap Q HP column (GE Healthcare, USA) as described elsewhere^34^. Active photoproteins and apo-obelin were eluted as separate peaks with the linear salt gradient of 1 M NaCl in 5 mM EDTA, 20 mM Tris–HCl pH 7.2. Protein concentration was determined with the Dc Bio-Rad protein assay kit.

### Crystallization, data collection, structure solution and crystallographic refinement

For Ca^2+^-discharged obelin-*v* to be obtained, an active obelin-*v* sample after ion-exchange chromatography was concentrated to 1.0 mg, using 10 kDa Amicon Ultra Centrifugal Filters (Merck Millipore, USA) with simultaneous replacement of chromatography buffer to a buffer containing 4 mM CaCl_2_, 20 mM Tris-HCl pH 7.2 at 4 °C. The Ca^2+^-discharged protein was then concentrated to ~ 10 mg/mL with the same Amicon Ultra Centrifugal Filters.

Protein crystals were obtained by the sitting drop vapor diffusion method in 96-well crystallization plates (SPT Labtech, UK). For screening initial crystallization conditions, the NT8 crystallization robot (Formulatrix, USA) and commercially available crystallization screening kits JCSG, PACT and SG1 (Molecular Dimensions) were used. Each sitting drop contained 200 nl of protein solution and 200 nl of reservoir solution. The best condition for crystallization of Ca^2+^-discharged obelin-*v* was a solution of 1.6 M Sodium citrate tribasic dihydrate (SG1 kit). Thereafter, the Ca^2+^-discharged obelin-*v* crystals were grown for 3–7 days at 20 °C to the final size of 100 μm. For X-ray diffraction analysis, the crystals were picked up from the crystallization drop using fiber loops and were flash-cooled in liquid nitrogen. Prior to freezing, the crystals were cryoprotected by transfer to a crystallization solution containing 20% vol/vol glycerol and soaking them for several seconds.

Data from the Ca^2+^-discharged obelin-*v* crystals were collected on beamline ID23-1 using Eiger2_16M detector at the European Synchrotron Radiation Facility (ESRF), France. Native diffraction data were indexed, integrated and scaled in P6_1_ space group using the XDS software^41^. The initial model was obtained by molecular replacement (MR) pipeline of Autorickshaw^42^ using the structure of active wild type obelin (PDB: 1EL4)^18^ as a search model. After that, the model was iteratively refined with PHENIX^43^ and adjusted manually using Coot^44^. Visualization and superposition of the molecular structures were performed using PyMOL 2.5.0 (Schrödinger, LLC). Parameters to detect hydrogen bonds were 3.6 Å for an ideal geometry and 3.2 Å for minimally acceptable geometry, 180° for a hydrogen bond cone, and 63° for the maximal hydrogen bond angle^45^. RMSD was calculated using Align method of PyMOL 2.5.0.

The spatial structure of Ca^2+^-discharged obelin-*v* was determined with a final resolution of 2.1 Å and deposited at PDB bank under ID 8A9S (Table 2).

**Table 2:** Data collection and refinement statistics for Ca^2+^-discharged obelin-*v*.

	Ca^2+^-discharged obelin-v*
Wavelength, Å	1.0332 Å
Resolution range, Å	32.66–2.1 (2.175–2.1)
Space group	P6_1_
Unit cell, Å, °	77.3 77.3 182.2 90 90 120
Total reflections	121,927 (12,305)
Unique reflections	35,579 (3561)
Multiplicity	3.4 (3.5)
Completeness, %	99.2 (98.9)
Mean I/σ(I)	10.1 (1.9)
Wilson B-factor, Å^2^	37.9
R_merge_, %	11.3 (147.0)
R_meas_, %	13.4 (174.6)
R_pim_, %	7.1 (93.1)
CC_1/2_, %	99.5 (39.9)
CC*, %	99.9 (75.5)
Reflections used in refinement	35,565 (3558)
Reflections used for R-free	716 (78)
R-work, %	16.0 (28.6)
R-free, %	21.0 (35.7)
**Number of non-hydrogen atoms**	3522
Macromolecules	3109
Ligands	138
Solvent	341
Protein residues	383
RMS (bonds), Å	0.003
RMS (angles), °	0.49
Ramachandran favored, (%)	98.94
Ramachandran allowed, (%)	1.06
Ramachandran outliers, (%)	0.00
Rotamer outliers, (%)	0.61
Clashscore	1.79
**Average B-factor, Å**	44.1
Macromolecules	44.4
Ligands	56.2
Solvent	47.9

*Statistics for the highest-resolution shell are shown in parentheses.

### Time-resolved fluorescence measurements

The Ca^2+^-discharged photoprotein samples for spectral measurements were prepared after the bioluminescence reaction ceased. Photoprotein samples were desalted on the HiTrap Desalting column with Sephadex G-25 resin (GE Healthcare, USA) equilibrated with 1 mM CaCl_2_, 20 mM Tris–HCl pH 7.2.

The absorption spectra of Ca^2+^-discharged obelins were measured using a Cary 5000 spectrophotometer (Agilent Technologies, Australia). Steady-state fluorescence spectra of the proteins were obtained with a Fluorolog-3 spectrofluorometer (Horiba Jobin Yvon, USA). The intensity was collected within the ranges of 380–750 nm and of 412–750 nm under excitation at 373 or 407 nm for Ca^2+^-discharged obelin and obelin-*v*, respectively. All fluorescence spectra were corrected for photomultiplier spectral sensitivity, inner filter effect and background signal.

Time-resolved fluorescence decays were obtained using a time-correlated single photon counting (TCSPC) method with DeltaHub module of Fluorolog-3 (Horiba Jobin Yvon, USA). A NanoLED N-370 with the peak wavelength at 373 nm and pulse duration of < 1.2 ns and DeltaDiode DD-405L with the peak wavelength at 407 nm and pulse duration of < 70 ps were applied for excitation for Ca^2+^-discharged obelin and obelin-*v*, respectively. Intensity decays were recorded within the ranges of 380–592 nm and 413–650 nm with a step of 2 nm and 3 nm correspondingly for Ca^2+^-discharged obelin and obelin-*v*; time resolution was 0.027 ns/channel.

To obtain time-resolved area-normalized emission spectra (TRANES), the following steps were performed^27^:

(i) In order to recover the fluorescence decay parameters (amplitudes and lifetimes), the sets of decays were deconvoluted from the pulse excitation response and fitted by a multi-exponential function using the global analysis approach^28^ with the DAS6 software (Horiba Jobin Yvon, USA). Time-resolved fluorescence decay at the wavelength λ was described as a sum of two (for obelin) or four (for obelin-*v*) exponents:

1 $${I}_{\lambda }\left(t\right)=E\left(t\right)\otimes {\sum }_{i=1}^{2or4}{\alpha }_{i}^{\lambda }{e}^{-\frac{t}{{\tau }_{i}}},$$

where *E(t)* is the instrument response function, *τ*_*i*_ is the lifetime and ${\alpha }_{i}^{\lambda }$ is the amplitude of the *i*-component of the lifetime at a wavelength *λ*. The fit quality was evaluated by its global χ^2^ value and weighted residuals.

(ii) Time-resolved emission spectra (TRES), plotted as intensity vs wavelength, were constructed using ${\alpha }_{i}^{\lambda }$ and *τ*_*i*_, and steady-state emission spectrum. The equation used was:

2 $$I\left(\lambda ,t\right)={I}_{ss}\left(\lambda \right)\frac{{\sum }_{i=1}^{2 or 4}{\alpha }_{i}^{\lambda }{e}^{-\frac{t}{{\tau }_{i}}}}{{\sum }_{i=1}^{2 or 4}{\alpha }_{i}^{\lambda }\cdot {\tau }_{i}},$$

where *I*_ss_(*λ*) is the steady-state fluorescence intensity at *λ*.

(iii) Finally, TRANES were constructed by normalizing the area of each TRES so that the area of the spectrum at time *t* was equal to the area of the spectrum at *t* = 0.

Decay-associated spectra (DAS) were obtained as follows:

3 $${I}_{i}^{\lambda }={f}_{i}^{\lambda }{I}_{ss}\left(\lambda \right),$$

where ${f}_{i}^{\lambda }$ is the contribution of each lifetime component to fluorescence at wavelength *λ*:

4 $${f}_{i}^{\lambda }=\frac{{\alpha }_{i}^{\lambda }{\tau }_{i}}{{\sum }_{j=1}^{2 or 4}{\alpha }_{j}^{\lambda }{\tau }_{j}}.$$

## Supplementary Information

Supplementary Information.

## Data Availability

All crystallographic coordinates and structure factors have been deposited in the PDB under the accession code 8A9S. All other data are available from the corresponding author on reasonable request.
