# Supplementary material for: Crystal structure of semi-synthetic obelin-v after calcium induced bioluminescence implies coelenteramine as the main reaction product
Source: Sci Rep. 2022 Nov 15;12:19613. doi: 10.1038/s41598-022-24117-5 (PMC9666459; doi:10.1038/s41598-022-24117-5)
Supplement: Supplementary file 1 — Supplementary Information. [file 41598_2022_24117_MOESM1_ESM.pdf]

SUPPLEMENTARY INFORMATION

Crystal structure of semi-synthetic obelin-*v* after calcium induced bioluminescence implies coelenteramine as the main reaction product

Pavel V. Natashin[1], Elena V. Eremeeva[1,2], Mikhail B. Shevtsov[3], Margarita I. Kovaleva[3], Sergey S. Bukhdruker[3], Daria A. Dmitrieva[3], Dmitry V. Gulnov[2], Elena V. Nemtseva[1,2], Valentin I. Gordeliy[4,5], Alexey V. Mishin[3], Valentin I. Borshchevskiy[3,6,*], Eugene S. Vysotski[1,*]

[1]Photobiology Laboratory, Institute of Biophysics SB RAS, Federal Research Center "Krasnoyarsk Science Center SB RAS", Krasnoyarsk, Russia

[2]Institute of Fundamental Biology and Biotechnology, Siberian Federal University, Krasnoyarsk, Russia

[3]Research Center for Molecular Mechanisms of Aging and Age-related Diseases, Moscow Institute of Physics and Technology, Dolgoprudny, Russia

[4]Institut de Biologie Structurale (IBS), Université de Grenoble Alpes, CEA, CNRS, Grenoble, France

[5]Institute of Crystallography, University of Aachen (RWTH), Aachen, Germany

[6]Joint Institute for Nuclear Research, Dubna, Russia

Correspondence

Valentin I. Borshchevskiy, Research Center for Molecular Mechanisms of Aging and Age-related Diseases, Moscow Institute of Physics and Technology, Dolgoprudny, Russia

E-mail: borshchevskiy.vi@phystech.edu

Eugene S. Vysotski, Photobiology Laboratory, Institute of Biophysics SB RAS, Federal Research Center "Krasnoyarsk Science Center SB RAS", Krasnoyarsk, Russia

E-mail: eugene_vysotski@ibp.ru

Supplementary Figures

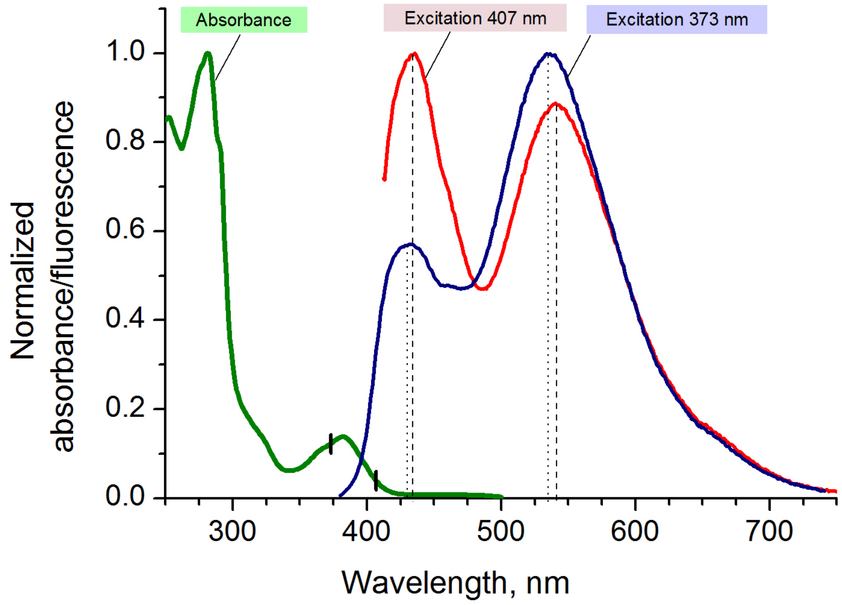

Fig. S1 Absorption (green) and fluorescence spectra under excitation at 373 nm (blue) and 407 nm (red) of Ca^{2+}-discharged obelin-*v*. Dashed and dotted lines refer to the apparent maxima of the emission spectra. The ticks on the absorption spectrum indicate the excitation wavelengths (373 nm and 407 nm).

Supplementary Tables

Table S1 Fluorescence lifetime components of Ca^{2+}-discharged obelin-*v* fluorescence under excitation at 373 nm

Ca^{2+}-discharged protein	Spectral range / Excitation wavelength	τ_i, ns	f_i, %	λ_i^{max}, nm	χ^2
Obelin-*v*	392–650 nm / 373 nm	0.33	6.2	419	1.09
		1.63	24.2	425	
		3.24	21.5	440, 525	
		8.07	48.1	547	

τ_i – lifetime component calculated by global analysis of the decays within indicated spectral range; f_i – spectral fraction of the component with τ_I in total emission spectrum; λ_i^{max} – maximum of the spectrum, associated with τ_I, (DAS$_i$); χ^2 – statistical criterion characterizing the quality of analysis using indicated lifetime components and their amplitudes.
